# Moxidectin use in Scottish sheep flocks suggests a need for clearer product labelling and communication of updated SCOPS guidelines

**DOI:** 10.1002/vetr.2083

**Published:** 2022-08-27

**Authors:** Jennifer McIntyre, Laura Miskell, Eric R. Morgan, Fiona Lovatt, Roz Laing

**Affiliations:** ^1^ School of Biodiversity, One Health and Veterinary Medicine University of Glasgow Glasgow UK; ^2^ Institute for Global Food Security Queen's University Belfast Belfast UK; ^3^ Flock Health Barnard Castle UK

## Abstract

**Background:**

Guidelines for sustainable use of moxidectin were established in 2020. This study aimed to identify how Scottish sheep farmers are using this key endectocide and estimate its effectiveness against gastrointestinal nematodes.

**Methods:**

Questionnaires were distributed to sheep farmers across Scotland, and analysis focused on moxidectin use in relation to Sustainable Control of Parasites in Sheep (SCOPS) guidelines. Farmers using moxidectin in their flock volunteered to submit post‐treatment sheep faecal samples, which were analysed for the presence of gastrointestinal nematodes using faecal egg counts with polymerase chain reaction to determine species.

**Results:**

Despite 70% of farmers using moxidectin in 2020, knowledge levels varied: 24% of farmers included other anthelmintics when asked about moxidectin use. Moxidectin was used for a wide variety of reasons, and most farmers did not consistently follow SCOPS guidelines. Despite only 2 of 76 farmers reporting failure of moxidectin treatment, gastrointestinal nematodes were found following moxidectin treatment on five out of six farms tested and included *Teladorsagia circumcincta*, *Cooperia curticei*, *Haemonchus contortus* and *Nematodirus* sp.

**Conclusion:**

Findings from this project indicate the need for improved anthelmintic product labelling and farmer support to encourage sustainable use. The presence of nematodes in treated animals is suggestive of anthelmintic resistance.

## INTRODUCTION

Moxidectin (MOX) is a milbemycin oxime, part of the macrocyclic lactone class of anthelmintics (group 3‐ML). It is a highly potent endectocide,^1^, ^2^ and the only anthelmintic to have persistent action against immature stages of two of the most pathogenic gastrointestinal nematode (GIN) species^3^: *Teladorsagia circumcincta*, an abomasal worm ubiquitous on UK farms,^4^ and *Haemonchus contortus*, which historically has occurred sporadically in the UK, but is anticipated to increase in distribution and prevalence due to climate change.^5^

Anthelmintic resistance (AR) is increasing^6^, ^7^ and the UK industry‐led initiative ‘Sustainable Control of Parasites in Sheep’ (SCOPS, www.scops.org.uk) provides guidance to vets, farmers and advisors on the best use of anthelmintics in order to prolong the efficacious life of a product on farm. Maintaining a population of sensitive GIN on a farm is facilitated by leaving some animals untreated when using a product, such that GIN within these untreated animals (in refugia from the drug) can seed the pasture with new eggs.^8^ MOX provides a unique challenge to refugia– due to its persistent activity, it takes a minimum of 5 weeks before sensitive GIN L3 of certain pathogenic species are able to re‐establish infection. The 2% long acting (LA) injectable formulation often employed^9^ has persistent activity against *T. circumcincta* for 97 days, and against *H. contortus* for 111 days, such that sensitive parasites fail to establish patent infection for 114–128 days following treatment.^10^ MOX resistance can present as ‘head’ resistance, when adults and/or L4 survive treatment, with less than 95% reduction in faecal egg count (FEC) 14–21 days post‐treatment.^11^, ^12^, ^13^ and as ‘tail’ resistance, when ingested L3 of certain species establish patent infection during the persistency period.^3^, ^14^ Further, when used to treat sheep scab, that is, psoroptic mange caused by the mite *Psoroptes ovis*, all sheep must be treated in order to extirpate mites and prevent rapid re‐infestation of the flock from untreated carriers.

To encourage sustainable use of MOX, SCOPS and the manufacturer Zoetis UK released a joint statement in January 2020 containing advice on treatment practices for all formulations.^15^ These are supplemented by other general and specific advice on the SCOPS website and in the technical manual,^16^ and are summarised in Box 1. The aim of this study was to understand how sheep farmers in Scotland are currently using MOX and whether they perceive any treatment failure. In addition, farms using MOX were asked to send two sets of samples following MOX treatment to the University of Glasgow to assess whether head or tail resistance might be present. Flock management data were collected using questionnaires, and FECs were followed by polymerase chain reaction (PCR) to determine parasite species. It is worth noting that species identification is essential to detect tail resistance—it cannot be confirmed from a strongyle FEC alone as L3 of only some strongyle species are sensitive to MOX.

## METHODS

### Questionnaire design and dissemination

Three questionnaires (described below) were designed to gather information during 2021 regarding MOX use by Scottish sheep farmers in 2020 and to identify whether they suspected the presence of AR on their farm. All questionnaires were sense checked by experts in the field, which included veterinary professionals, a sheep veterinary consultant and a researcher experienced in knowledge exchange with farmers. Both paper and online formats were used to encourage as many farmers as possible to take part, and were a collaboration between two separate studies to reduce farmer questionnaire fatigue. Questions focusing on MOX use are described and analysed in this study. Data collected from eight questions, present in all three questionnaires, were combined for a descriptive analysis.

**Box 1: SCOPS moxidectin principles**

- Avoid using moxidectin (MOX) when aiming to treat only fluke, scab or *Nematodirus*—use a narrow spectrum product instead.
- ‘Dose, delay and move’ will not work to preserve MOX‐susceptible gastrointestinal nematodes, due to its persistent action against *Teladorsagia circumcincta* and *Haemonchus contortus*. Instead, use targeted selective treatment (TST)/treat less than 100%.
- MOX 2% long acting: Do not use this formulation more than once in a flock during a 12‐month period for any reason (e.g., do not use it in March in ewes to treat the peri‐parturient rise [PPR], and again in October to treat sheep for scab).
- MOX 1% injection: If using to treat scab, give two injections, 10 days apart.
- When treating sheep scab (*Psoroptes ovis*), treat all sheep. In contrast, when treating worms, aim to leave some sheep untreated in each grazing group to maintain susceptible worms in refugia.
- Ewe PPR: If treating with MOX, use a different anthelmintic the following year.
- PPR (any anthelmintic, including MOX): Leave at least 10% of ewes untreated in any grazing group. Choose those to leave untreated using TST indicators (e.g., body condition score).
- Always check the dose rate and administration method and do not under‐dose.

A paper version (nine questions, Supporting Information S1) was distributed via sheep technical advisors/farming merchants and 7000 copies were sent out in the Scottish Farmer, a popular farming publication, with FREEPOST reply envelopes included also. Questions used in this study asked for details of the farm location, flock size, use of MOX in 2020 and the farmers intended future use of MOX. In addition, farmers were asked about their beliefs concerning the treatment of the peri‐parturient rise (PPR), their use of the amino‐acetonitrile derivative (ie, group 4‐AD) and spiroindole (ie, group 5‐SI) classes of anthelmintics and their perception regarding the presence or absence of AR on their farm. A longer online version (‘WormScot’), containing the same nine questions with a further 18 additional questions covering SCOPS management practices more broadly, was primarily distributed via Twitter and Scottish farming organisations. Care was taken to ensure that identical questions would be interpreted in the same way in either version. MOX use was answered using free text (paper questionnaires) or via selection from drop‐down menu options (online questionnaire ‘WormScot’). It is important to note that the question had identical wording and examples in both versions. All other questions included in this study were dichotomous or multiple choice, excluding the farm location which was free text.

A separate questionnaire (paper format, Supporting Information S2) was answered only by farmers who submitted sheep faecal samples for the study. This contained four main sections covering: treatment and sample collection related to the samples submitted, general worm control practices, MOX use and AR. There were 34 questions, 26 of which were dichotomous or multiple choice (with three allowing further explanation of an answer choice), six allowed free text but were closed ended and two used an unmarked semantic differential scale (Supporting Information 2).

### Sheep faecal sample collection

The project was widely advertised using posters, emails, Facebook posts, tweets and an article in the Scottish Farmer. Farming merchants, vets and sheep organisations assisted in dissemination of advertising material. Six farmers across Scotland using MOX in their flock subsequently, and voluntarily, contacted the researchers using email or by phone, in accordance with current General Data Protection Regulation (GDPR) regulations in the UK.

Farmers were asked to collect individual faecal samples from sheep at two time points during 2021. These will be referred to hereafter as the ‘post‐MOX sample’ (requested 14–21 days post‐treatment from 15 sheep) and the ‘persistency period sample’ (requested 28–48 days following oral or 1% injectable treatment or 90–111 days following 2% LA injectable formulation from 10 sheep). The post‐MOX sample tested for ‘head’ resistance—adults and/or L4 surviving treatment. The persistency period sample tested for ‘tail’ resistance—establishment of new, patent, infection by L3 of *T. circumcincta* or *H. contortus*.^10^, ^13^, ^17^

Anticipating that the FEC of the post‐MOX samples would be low, or zero, samples collected from 15 individuals were requested to increase confidence in a low egg count. As higher numbers of faecal eggs were expected to be present in the persistency period samples, only 10 samples were deemed necessary, reducing labour in sample collection. Sample kits and collection guidelines were provided (Supporting Information S3). In particular, clear instructions were provided to ensure that faeces were stored anaerobically to prevent egg hatching, and, where possible, kept at ambient temperature to allow larval culture after arrival in the laboratory. If samples were unlikely to arrive within 48 hours of collection, farmers were asked to place faeces at 4°C. Farmers were asked to specify how samples were stored following collection and whether (and how) they had deviated from the instructions (Supporting Information S2). Samples were processed on the day of arrival.

A total of 12 faecal samples were received from six farmers within 48 hours of collection for all but two samples; one was delivered within 72 hours of collection and had a degree of development within the eggs, including a few larvated eggs (Farm 4, second sample), the other (Farm 5, second sample) was stored in a fridge on the same day as collection, was collected for delivery and returned to the farmer, and remained in a fridge while collection was re‐organised. It was re‐collected on day 8 following collection and was received within 24 hours—the eggs were still in the blastomere stage.

### Lab Methods

#### Faecal egg count and coproculture

To count faecal eggs, a cuvette method, sensitive to 1 egg per gram (epg) was used based on the method of Christie and Jackson.^18^ Briefly, median 4.2 g faeces (minimum 2.1 g due to small sample) was weighed and water was added in a ratio of 10 ml to 1 g, following which faeces were homogenised, strained through a 250 μm aperture sieve and a further 5 ml water per 1 g faeces was added. After centrifugation of 15 ml at 1061 × *g* for 5 minutes (Centaur 2 MSE), the supernatant was discarded and the egg pellet was re‐suspended in saturated sodium chloride solution (specific gravity 1.2) with a further centrifugation step at 170 × *g* for 10 minutes. Centrifugation parameters were those routinely used for diagnostics at the University of Glasgow laboratory. The meniscus was isolated using artery forceps and tipped into a cuvette, which was filled with saturated NaCl solution for counting.

To culture larvae, pooled faeces were mixed with vermiculite and incubated at 25°C for 10–12 days before Baermannisation. Briefly, a Büchner funnel was used to transfer larvae and debris to Whatman Grade 113 filter paper under vacuum suction. The filter paper was subsequently upturned onto a milk filter (DairyCo) and floated on tap water at room temperature in a glass funnel to allow viable larvae to migrate downwards. Debris and dead larvae were retained by the filter. After 3 hours, L3 were collected with a rubber exit tube at the base of the funnel. Larvae were stored at 8°C for short term, and frozen at –80°C for long‐term storage. If faeces had been stored at 4°C, eggs were harvested from the remaining FEC filtrate and those collected were used for PCR.

#### Identification of strongyle species by PCR

DNA lysates were made from individual eggs (six samples, see Table 1 for details) or L3 (six samples) and species identified by single or multiplex PCR as described by McIntyre et al.^19^ For the post‐MOX sample, PCRs were performed to detect seven strongyle species (*Chabertia ovina*, *Cooperia curticei*, *H. contortus*, *Oesophagostomum venulosum*, *T. circumcincta*, *Trichostrongylus axei* and *Trichostrongylus vitrinus*). Up to 96 strongyles were identified by PCR for each sample, where sufficient material was present (see Table 1). For samples collected during the expected period of MOX persistency (‘persistency period samples’), PCRs were only performed to identify the presence of *T. circumcincta* and *H. contortus*, as these are the only species against which persistent activity is claimed for the full time period studied.

**TABLE 1:** Faecal sample results

		Post‐MOX sample (2–3 weeks post treatment)			Persistency period sample (6 or 13 weeks post‐treatment)		
Farm	Formulation used	Days post‐treatment	Mean faecal egg count (epg)^a^	Species identified by PCR (*n* species/*n* strongyles)	Days post‐treatment	Mean faecal egg count (epg)^a^	Species identified by PCR
1	2% LA	13	0 (0)	None^b^	91	36 (32)	None
2	2% LA	19	11 (6)	*Teladorsagia circumcincta* (50/96)	88	576 (419)	None
				*Cooperia curticei* (46/96)			
3	MOX/triclabendazole combination	13–19	16 (16)	*T. circumcincta* (74/74)	40–45	73 (67)	None
4	Oral	14	71 (100)	*T. circumcincta* (82/82)	42	230 (214)	None
5	2% LA	22	7 (4)	*T. circumcincta* ^c^ (2/2)	100	261 (128)	*T. circumcincta* ^c^
6	Oral	17	4 (2)	None	41	15 (27)	*T. circumcincta*
							*Haemonchus contortus*

*Note*: Species were identified by polymerase chain reaction (PCR); for the persistency period sample PCR was only performed to identify *T. circumcincta* and *H. contortus*, and only when these species had not been identified in the post‐moxidectin (MOX) sample. Life‐cycle stages used for PCR were as follows (Farms 1–6): for post‐MOX samples—none, L3, eggs, L3, eggs, none; for persistency period samples—L3, L3, eggs, L3, eggs, L3.

Abbreviation: epg, egg per gram; 2% LA, 2% long acting injectable; moxidectin, MOX.

^a^ Rounded to the nearest whole number. Standard deviation (SD) given in brackets.

^b^ No eggs or larvae were available for PCR, however samples from four sheep contained strongyle eggs suggestive of *C. curticei* by morphological appearance.

^c^ Only a small number of eggs from the post‐MOX sample were available for PCR, of which only two were positive for *T. circumcincta* (the others failed to amplify) —for this reason, the persistency period sample was also tested for *T. circumcincta*.

### Statistical analysis

Anonymised questionnaire data were pooled for analysis when questions were identical between the three questionnaires used (Supporting Information S1, ‘WormScot’ and Supporting Information S2). Answers were coded where appropriate and analysed descriptively using Microsoft Excel and R v 3.6.3. When appropriate, proportion tests [prop.test()] were performed. FECs were analysed using arithmetic means, and Wilcoxon rank sum tests were performed in R. Graphs were plotted in R v 3.6.3, using ggplot2 v 3.3.0 and scales v 1.1.0.

## RESULTS

### Farmer response

In total, 104 questionnaires were returned, of which 76 were from sheep farms in Scotland. Most questionnaires were returned using FREEPOST addressed envelopes distributed via the Scottish Farmer (81%). Additional responses were obtained via various merchants and sheep advisors across Scotland (6%) and using an online survey ‘WormScot’ (7%). Farmers who had sent in faecal samples completed a separate questionnaire and, from this, answers were extracted for questions, which were identical to those in the primary questionnaire (6%). Questionnaires completed by farmers located outside of Scotland, or who did not specify a location, were excluded from this study.

**FIGURE 1:**
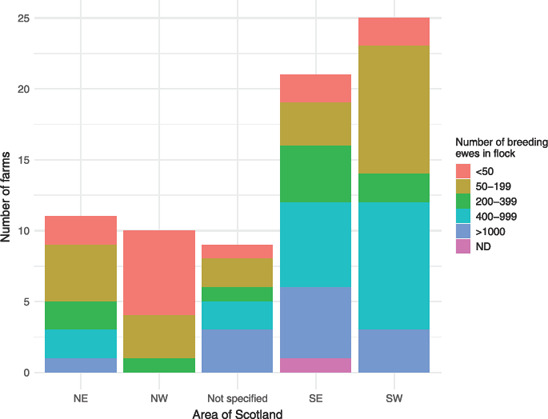
Number of farms completing questionnaires by region in Scotland, coloured by number of breeding ewes. ND, no data

Responses were obtained from across Scotland, with more farmers in the south responding than in the north (Figure 1). Nine Scottish farmers did not specify their county. A greater proportion of farms in the south had 400 or more breeding ewes compared with the north, and none with this number responded from the northwest. In contrast, although farms with fewer than 50 breeding ewes were represented in all regions, 46% were located in the northwest, representing 60% of the farms that responded from this region (Figure 1).

### Knowledge of moxidectin products varied

Farmers were asked to report all uses of MOX in their flock during 2020, providing the product/formulation, sheep group, month treated, proportion treated and reason for use. Where farmers had specified simply ‘Oral drench’, this was assumed to mean the MOX oral formulation, rather than a separate anthelmintic. Of those responding using paper questionnaires, which allowed free text, 11% positively identified that they had not used MOX in 2020. In contrast, 24% wrote down anthelmintics other than MOX, even though the question specifically sought information on MOX products. Several farmers included these alternative anthelmintics alongside MOX products, while others did not report any MOX products. Reasons given for MOX use included five instances that implied misunderstanding of the product or parasite epidemiology, including the use of the oral formulation of MOX to treat sheep scab, treating fewer than 100% of sheep for scab and using a broad‐spectrum combination product (MOX and triclabendazole) to treat lambs and ewes for fluke in spring.

### Reasons for moxidectin use

**FIGURE 2:**
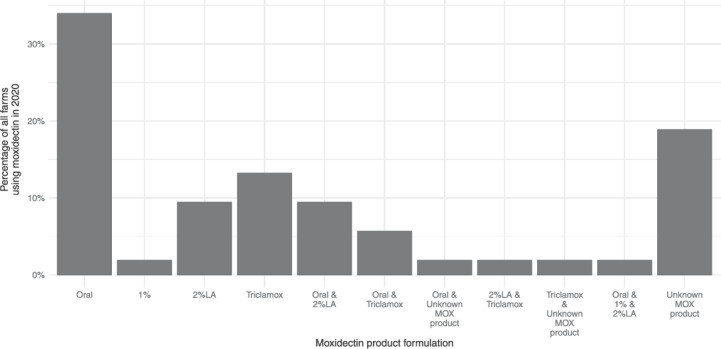
Moxidectin (MOX) products used in 2020 by farmers. Percentage of 53 farmers that used moxidectin in 2020 is shown. Unknown MOX product: Brand name listed, but not formulation. 2% LA, 2% long acting injectable

**FIGURE 3:**
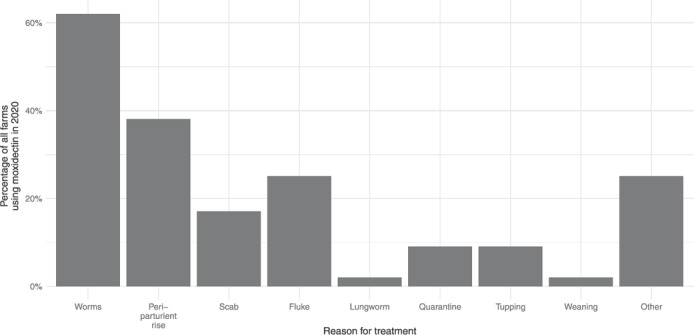
Reason given for treatment using moxidectin in 2020 by farmers, grouped during analysis into nine categories. Percentage of 53 farmers that used moxidectin in 2020 is shown.

Seventy percent of farmers (53) reported that they had given at least one MOX treatment in 2020 (Figure 2), providing a broad range of reasons for use. These were condensed into nine categories: worms, PPR, scab, fluke, lungworm, quarantine, tupping, weaning and other (Figure 3). Thirty farmers listed more than one reason for using MOX during 2020, although not necessarily for any given treatment. ‘Worms’ was the most common reason provided, with 62% of 53 farmers using it for this purpose. Those specifically indicating that they had used MOX as a ewe treatment to counteract the PPR in 2020 amounted to 38%.

Quarantine purposes included both bought‐in sheep and those which had overwintered off farm. Tupping (9%) and weaning (2%), in addition to various ‘other’ reasons (25%), suggested that MOX was used for routine treatments on many farms and not necessarily when most appropriate for parasite control. In addition, ‘worm’ treatments were often coupled with a management event, suggesting that treatments were given when convenient, rather than targeted.

### Compliance with SCOPS principles

SCOPS provide advice on MOX use for both worms and scab, and in particular for the PPR (Box 1). Farmers are advised not to use it for the PPR in consecutive years and to treat not more than 90% of ewes. More generally, SCOPS advocates for anthelmintic use, which preserves parasites in refugia, to maintain sensitivity within the farm parasite population to anthelmintics in the future. This includes using the right product at the right time, and selectively treating within a group of sheep so that some are left untreated.

Of the 53 farmers using MOX in 2020, 10 had used it only when treating for scab, fluke, quarantine purposes or ‘other reason’ (the latter was an online response chosen from a drop‐down menu and was interpreted as use for fluke in addition to worms). Of the remaining 43 farmers, 12 (28%) had treated fewer than 100% of sheep in the treatment group on at least one occasion. Of these, only eight had treated 90% or fewer animals within a group, with a ninth farmer not specifying a percentage but stating they treated ‘those with dirty rears’. Only two farmers treated fewer than 100% of sheep in a group on all occasions during 2020; one used this approach once and the other twice. Of those farmers using MOX for the PPR in 2020, just three (15%) left at least one in 10 ewes untreated.

**FIGURE 4:**
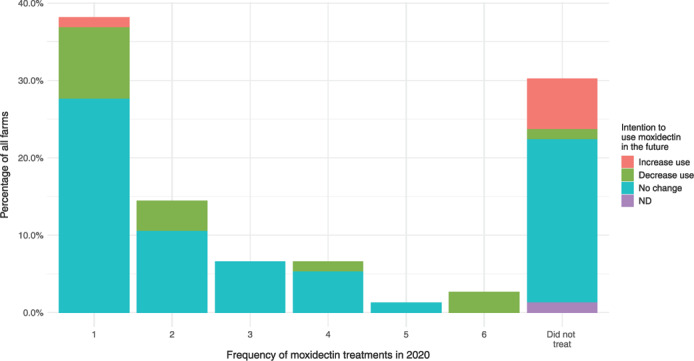
Intention to change moxidectin use shown against the frequency of moxidectin treatments given in 2020 by farmers. ND, no data

To determine the frequency of MOX use in 2020 by Scottish farmers, responses were grouped by time and treatment decision. For example, if more than one age group was recorded as being treated for fluke in a given month, these responses were grouped together to form a single ‘treatment’. Similarly, if ewes were recorded as treated for the PPR in March and April, this was recorded as a single treatment. Just over half the farmers using MOX in 2020 (54%) used it once, of which 41% treated only ewes. Overall, 85% of 53 farmers using MOX treated ewes with MOX in 2020, and 25% treated only ewes. A fifth of famers used MOX twice within their flock; the remaining 25% used it on three to six occasions during the year. Seventy‐two percent of 76 farmers stated that they did not intend to change their MOX use in the future (Figure 4). Six farmers intended to increase their MOX use, of which five had not used it during 2020. Of 14 farmers intending to decrease MOX use, seven had used it just once.

While it is advised to treat less than the entire group of sheep for worms, to preserve anthelmintic efficacy via refugia, it is appropriate and necessary to treat the entire group for sheep scab (*P. ovis* ^16^). MOX injectable formulations can be used for this purpose. Nine farmers administered MOX during 2020 to treat or prevent sheep scab. Six used it according to SCOPS guidelines; two treated less than 100% of the group and one used an oral formulation. SCOPS advocates using the 2% LA formulation not more than once per annum. One farmer, who had treated fewer than 100% of the group, reported use of the 2% LA formulation twice for scab during 2020. Nevertheless, apart from reported use for quarantine purposes, no other farmers used the 2% LA formulation more than once in 2020.

Liver fluke, *Fasciola hepatica*, has a complex life‐cycle and management, which includes treating with flukicides during autumn, winter and spring. As acute fluke infection can cause considerable morbidity and mortality in sheep,^20^ it is often necessary to use a triclabendazole product in the early stages of infection. Although MOX does not treat liver fluke (*F. hepatica*), CYDECTIN TriclaMox (Zoetis), containing both triclabendazole and MOX, is available for sheep. Triclabendazole is the only anthelmintic that is active against immature stages of liver fluke, but resistance against this drug is increasing.^21^ SCOPS advises solely using triclabendazole for immature fluke during the autumn and early winter, employing other flukicides during late winter and spring. Eleven farmers specifically reported using TriclaMox (triclabendazole and MOX) during 2020. Of these, nine treated sheep during the autumn or winter, with the remaining two treating in March or May. A further four farmers reported treating for fluke, of which one used ‘fluke and worm drench’, another ‘Cydectin’ and two ‘oral drench’. Two of these farmers treated in March or May. Interpreting all 15 farmers to have employed TriclaMox, five used it solely for fluke.

### Farmer ideas and behaviour

**FIGURE 5:**
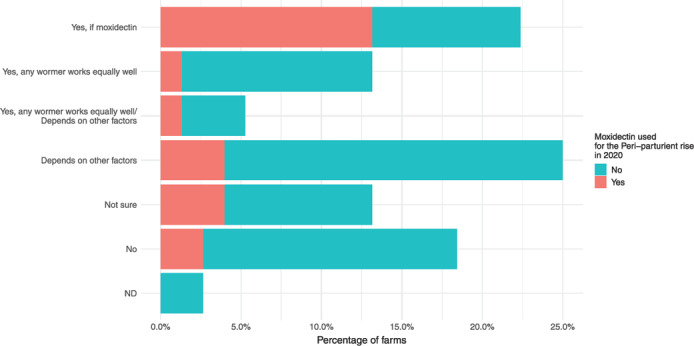
Farmer beliefs surrounding the benefit to lambs of treating ewes for the peri‐parturient rise, coloured by their moxidectin treatment behaviour for the peri‐parturient rise in 2020. ND, no data

All farmers were asked whether they felt that lambs performed better if ewes were treated at lambing time. Responses were roughly equally divided between all five answer options (Figure 5). Farmers who believed that MOX treatment of ewes during the PPR benefitted lambs (17 farmers) were more likely to have treated in 2020 (10 farmers) compared with those who did not hold this view (*p* < 0.05). Fourteen farmers did not believe there was a benefit to lambs of treating the PPR, yet two farmers who held this view still treated ewes with MOX at lambing time in 2020.

Farmers who submitted faecal samples for testing were asked in more detail about their worm management, in particular their MOX use. Although the number responding was very small (*n* = 6), all were aware of the need to calibrate their dosing equipment—four used a dosing gun, which was either new or had been calibrated within the last month (two farmers) or year (one farmer). In contrast, five used weigh scales but only three had calibrated them within the last year, one longer than a year ago and one had never done so. Asked whether they were concerned about 3‐ML resistance on their farm, three farmers scored their concern ≥3.5/5 (on an unmarked semantic differential scale). Unlike those who were less concerned about AR, all three stated that they were actively trying to maintain refugia of GIN on their farm that were sensitive to wormers. Nevertheless, out of the six farmers, only one treated fewer than 90% of ewes at lambing time, also mixed treated and untreated ewes following lambing, and they were unconcerned about AR (score 1/5, Farm 6). The other five farmers treated 98%–100% of ewes. Four farms had fields grazed by ewes which were treated with MOX at both the start and the end of the lambing period, prolonging selection pressure. All six farmers expressed a desire to use alternative methods to control worms in the future; pasture management, breeding and other livestock/livestock rotation were selected. Only one wanted to manage worms with anthelmintics in the future, and this was in addition to other management methods.

### Anthelmintic resistance—Perception and presence

All farmers were asked whether anthelmintics were working effectively on their farm and to select anthelmintic classes that they thought had reduced efficacy on their farm. These included all five broad‐spectrum anthelmintic classes, with the 3‐MLs differentiated into avermectins and MOX. Just two out of 76 farmers (one of which was Farm 1), suspected resistance to MOX on their farms, while 40 farmers (53%) did not perceive issues with any anthelmintics (including Farms 2–4). The commonest class with suspected AR was benzimidazoles (ie, group 1‐BZ), with 25 farmers (33%) thinking they had reduced efficacy. Eight farmers felt there was reduced efficacy to levamisole (group 2‐LV), seven to avermectin products, one to monepantel (group 4‐AD) and one to abamectin‐derquantel (group 5‐SI).

**FIGURE 6:**
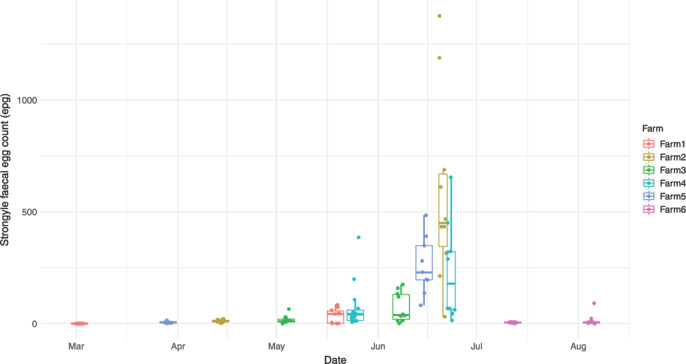
Strongyle faecal egg counts post‐treatment. Collected 2–3 weeks post‐treatment and again at the end of the persistency period of the product. Dots represent individual samples.

Post‐MOX samples were collected by farmers 13–22 days following treatment. Mean strongyle FECs of these samples were very low (0–16 epg, Table 1, Figure 6) on all but Farm 4, which had a mean of 71 epg. The persistency period samples were collected 40–45 days after oral treatment or 88–100 days after use of the 2% LA injection (Table 1). The latter is licensed to persistently prevent re‐infection with *T. circumcincta* for 97 days and *H. contortus* for 111 days following treatment, while the oral formulations claim to prevent re‐infection for 35 days for both parasites. Therefore, neither parasite should have been detected at either time‐sampling point. However, *T. circumcincta* was detected in the post‐MOX sample on four farms (confirmed using the persistency period sample on Farm 5, as the post‐MOX FEC was so low [7 epg] that only two *T. circumcincta* were identified by PCR). Farm 1 had neither *T. circumcincta* nor *H. contortus* detected in either sample, despite using MOX three times during 2020. Farm 6 post‐MOX sample had mean 4 epg and no strongyle eggs or L3 were available for PCR. In spite of a low FEC (mean 15 epg), both *T. circumcincta* and *H. contortus* were detected in the persistency period sample on Farm 6.

On two farms, *C. curticei* eggs, more distinctive than other ‘strongyle’ eggs, were noted in the post‐MOX sample during egg counting. The presence of this species was confirmed by PCR on Farm 2, but due to the failure of the few remaining eggs from Farm 1 to amplify, was not confirmed on this farm. *C. ovina*, *O. venulosum*, *T. axei* and *T. vitrinus* were tested for by PCR for all post‐MOX samples but were not identified on any farm. Strongyle species other than *T. circumcincta* and *H. contortus* were not tested for in the persistency period samples as the products do not claim a persistent activity against L3 of other species to the point of sampling. Three farms had *Nematodirus* eggs present in the first faecal sample and all three, along with a fourth farm, had *Nematodirus* in the second sample. For all farms, except Farm 6, the persistency period sample FEC was significantly higher than the post‐MOX sample by Wilcoxon rank sum test (*p* < 0.05).

## DISCUSSION

MOX is the only anthelmintic with persistent activity against re‐infection by certain pathogenic GIN species, and as such, selection for resistant parasites is expected to be prolonged in comparison to other sheep anthelmintics^14^. Sustainable guidelines for MOX use (Box 1) were released by SCOPS, in collaboration with the manufacturers Zoetis, in 2020^15^ and this study sought to assess how MOX was being used in Scotland, and whether these guidelines were followed. Seventy percent of Scottish farmers that responded used MOX in 2020. Although a small study, this research has shown that knowledge regarding MOX is highly variable among farmers in Scotland and that, with the exception of two individuals, all farmers treated all sheep at least once when using MOX for nematode control in 2020. This is in contradiction of SCOPS recommendations^15^ and indicates limited uptake of targeted selective treatment. Almost one in four farmers completing a paper questionnaire appeared confused over which products contained MOX, and mentioned other anthelmintics when asked about MOX. In addition, inappropriate use was evident in the responses, including using the wrong product (i.e., using oral MOX to treat sheep scab). Nevertheless, only one farmer reported using the 2% LA formulation more than once during 2020, other than for quarantine purposes. A limit of the study was the small sample size for the FECs and species data, in particular the post‐treatment sampling. It is therefore important to recognise that these results may not be representative of the wider situation in Scotland.

MOX is a key endectocide^1^, ^2^ and triclabendazole is an essential flukicide.^22^ Yet both MOX^23^, ^24^ and triclabendazole^21^ resistance are present in the UK, and prevalence is increasing in many countries.^7^, ^25^ Triclabendazole‐only products should be encouraged in adult animals, which are unlikely to require worming for nematodes in the autumn or early winter,^26^, ^27^ while organophosphate dips should be used for scab treatment where possible and when concurrent worming is not desired.^16^ This study found almost all MOX treatments were given to all animals in a group. While sometimes this is necessary (e.g., to treat scab), the recommended position for wormers is to leave a percentage of animals untreated to maintain GIN in refugia.^8^, ^16^ Where ewes graze alongside lambs, the impact of treating all lambs may be mitigated by leaving some ewes untreated. However, immunity in ewes is re‐established in the first 6 weeks or so following lambing when treatment is not given,^28^ and the ability of immune competent ewes to seed the pasture with sensitive parasites of *T. circumcincta* is reduced.^29^ Due to the persistent action of MOX against *T. circumcincta* and *H. contortus*, it is anticipated that AR might develop more rapidly than for short acting compounds. MOX is highly lipophilic and passes into the milk, such that selection for resistant parasites may also occur in lambs if their dams are treated with MOX for the PPR.^30^ MOX has efficacy against ivermectin‐resistant GIN,^31^ yet research suggests that once ivermectin resistance is present, MOX resistance establishes much more quickly than on a farm with ivermectin‐sensitive GIN, and ivermectin resistance itself increases.^32^ Careful use of MOX, including steps to ensure adequate refugia, is consequently highly desirable.

Detection of MOX‐resistant GIN in this study involved post‐treatment FECs and species identification only. Without pre‐treatment FEC information, the FEC reduction cannot be calculated. However, Farms 1–5 were treated at lambing time for the PPR, when egg counts were expected to be high. Although the study relied on farmers treating sheep correctly, the negligible FECs 2–3 weeks post‐treatment on four of these five farms suggested correct treatment procedures. It is likely that FEC reduction would have been above the 95% required to classify treatment as effective on these farms, but this would require a full FEC reduction test to confirm and ‘head’ resistance is suspected on Farm 4 in particular (mean 71 epg). In any study where exposure to infection is not controlled (i.e. all field studies) then it is possible that eggs detected post‐MOX may be from re‐infection when the pre‐patent period is shorter than 21 days. On Farm 1 neither *T. circumcincta* nor *H. contortus* were ever detected, despite use of MOX three times in the previous year. In contrast, the presence of *T. circumcincta*, and in one case *H. contortus*, in samples before the end of the persistency period on the other five farms should be cause for concern.^3^, ^23^, ^33^ Given the negligible egg counts at 2–3 weeks post‐treatment, this suggests that either some adult worms survived treatment but egg laying was temporarily suppressed^34^, ^35^ or that ingested L3 from pasture were able to establish new infections while the drug should still be active (‘tail’ resistance).^14^ More research is needed to understand how tail resistance relates to head resistance, and how quickly head resistance is likely to arise once ingested L3 can establish patent infection during the persistency period. In addition, it is important to determine how adults, which survive treatment in small numbers or which experience suppression of egg output (FEC reduction >95%), contribute to resistant genotypes in subsequent generations of GIN.

Treating ewes for the PPR can reduce larval challenge on pasture.^36^ Although there may be some benefit to the ewe herself, this effect may be small and other factors are also important.^37^ On many farms, treatment of the PPR might have limited benefits for either the ewe or her lamb.^38^ Despite this, four in 10 farmers believed that treatment of the PPR was of benefit to the lambs, and 38% of 53 farmers used MOX for the PPR in 2020. A recent study, considering all anthelmintic classes, found that 57% of 149 UK farmers treated ewes for the PPR.^39^ Interestingly, in the current study, not all farmers who felt that lambs performed better if ewes were treated with MOX had used it for this purpose in 2020. This might indicate that they are following SCOPS guidelines—not using MOX year‐on‐year for the PPR. Others felt that there was no benefit to lambs if ewes were treated, yet had still used MOX for the PPR in 2020. It could be that experience of treatment of the PPR with (or without) MOX in 2020 led farmers to formulate a belief of its impact on their lambs; however, it is also possible that prior belief motivated treatment practices rather than being a response to observed outcomes. As selection pressure by anthelmintics on GIN in the spring is more extreme than later in the year due to cold weather and low numbers of viable L3 on pasture,^40^ with further amplification of survivors of treatment through the grazing season, treatment decisions for the PPR are critical when thinking about maintaining refugia. It would be useful to follow up these findings with a more detailed study of Scottish farmers to identify why they hold their views, and, in particular, why they may still treat even if they do not believe it benefits lambs. Additional epidemiological studies are also needed to quantify impacts of treatment during the PPR on infection levels in lambs and on refugia, in interaction with climate and weather.

SCOPS produces a farmer‐friendly anthelmintic product guide, containing brand names alongside generic drug names. However, due to the typically large number of different commercial products for a single anthelmintic, most ‘best practice’ guidelines name the active anthelmintic ingredient only. Information relevant to a commercial product, bought by a farmer, may not therefore be easily recognised or remembered. In light of apparent confusion surrounding the contents of anthelmintic formulations used by 24% of the farmers in this study and the recommended application of MOX products, it is important to make sure that clear, visual information is present both at the point of sale and when farmers treat livestock. Improved labelling of products may help farmers use anthelmintics appropriately. In the UK, sheep anthelmintics (and others) may be voluntarily labelled with a symbol to show the anthelmintic class,^41^ with the aim to improve sustainable use. However, this study would suggest that this labelling scheme is not sufficient for many to understand which active anthelmintic ingredient is in the product they are using. Similar confusion over product ingredients and use occurs for flukicides (Williams D, personal communication, 2022).

Unifying packaging information about anthelmintic ingredients and parasites treated across all brands and products using simple pictorial information, which could also be displayed alongside SCOPS guidelines, may help with sustainable use. Research into what UK farmers understood from anthelmintic packaging and how they would prefer information to be presented would be useful to inform how this information could be most clearly and simply displayed.

Respondents with correct worm control knowledge and an agricultural education were associated with an increased likelihood to test for AR in a recent Scottish survey of 400 farmers.^43^ Confirmation of AR within this group was the strongest moderator of SCOPS behaviour, and the factor most likely to reduce the presence of AR selective behaviours.^42^ It is therefore important that (1) SCOPS advice is disseminated as widely as possible, and that (2) farmers are encouraged to test for AR. Yet, Morgan et al.^44^ found that only 13% of 600 UK farmers had heard of SCOPS and only 58% these 67 respondents could correctly state one guideline. In Belgium, dairy cattle farmers were found to be strongly influenced by their vet regarding worm control practices.^45^ UK studies find that farmers value vet advice regarding appropriate worm control—one Scottish study found that greater than 73% of 400 farmers felt vet advice on worm control was reliable or cost effective,^43^ but such advice is often sought elsewhere; including suitably qualified persons (SQPs), the farming press and other farmers.^44^, ^46^ Many UK sheep farmers develop their own worm control plans, without outside help, although they may gather advice from many external sources and choose what they deem relevant.^47^, ^48^ Melville et al.^39^ reported that just 12% of 149 UK farmers based lamb ‘strongyle’ GIN treatments on vet advice, and just 15% based ewe treatments on vet advice. Indeed, a survey of 325 UK farmers found that 24 purchased anthelmintics from their vet.^46^ In contrast, 103 bought anthelmintics from an SQP, with the remainder choosing to use more than one prescriber type.^46^ Importantly, purchasing practices reflected, at least in part, the source of anthelmintic advice used by the farmer. It is important therefore that individuals prescribing anthelmintics have good worm control knowledge. A recent online survey, completed by 227 UK vets and 57 UK SQPs found farm animal respondents answered 57.5% of ‘best practice’ questions correctly and 86.6% of respondents trained in both farm animal and equine medications did so.^49^ *T. circumcincta* is ubiquitous on UK farms,^4^ and highly pathogenic, yet concerningly, just 59% of SQPs and 80% of vets identified it to be a pathogen of sheep.^49^ Going forward, communication of key facts and best practice guidelines needs to encompass both the end user and prescriber in order to encourage sustainable use of anthelmintics and effective worm control.^44^

To conclude, this study found that knowledge of MOX and its appropriate use was highly variable among sheep farmers across Scotland, and of 53 farmers that reported at least one treatment with MOX in 2020, only two treated fewer than 90% of sheep in the group on all occasions that they employed MOX for nematode control. Only two out of 76 farmers reported MOX resistance on their farm, yet positive *T. circumcincta* FECs within the period protected by MOX were identified on five out of six farms tested, and *H. contortus* was identified on one farm. Although 30% of farmers did not report any MOX use during 2020 in their flock, 22% of these intended to increase their use. Farmers were keen to use alternative methods in the future to control worms, yet appear to currently rely heavily on anthelmintics. Finally, more should be done to make product information and SCOPS guidelines as accessible and simple as possible.

## FUNDING

This work was funded by a KTN/BSAS Steve Bishop Early Career Award to Jennifer McIntyre. Jennifer McIntyre and Eric R. Morgan were also funded by a Biotechnology and Biological Sciences Research Council (BBSRC) strategic Lola (BB/M003949) and Laura Miskell by a Teagasc Walsh Research Scholarship. This research was funded in whole, or in part, by the Wellcome Trust [216614/Z/19/Z].

## CONFLICT OF INTEREST

The authors declare they have no competing interests.

## AUTHOR CONTRIBUTIONS

Jennifer McIntyre conceptualised and designed the study, performed data collection, analysis and wrote the manuscript. Laura Miskell assisted with questionnaire design and performed data collection. Eric R. Morgan assisted with data collection and the manuscript, and provided advice. Fiona Lovatt designed the study, assisted with data collection and provided detailed advice on the study execution and manuscript. Roz Laing designed the study and assisted with data collection, analysis, guidance and the manuscript.

## ETHICS STATEMENT

Ethical permission was obtained from the University of Glasgow MVLS Ethics Committee for all work performed. Specific advice was sought from the Data Management Team regarding GDPR.

## Supporting information

Supplementary material

Supplementary material

Supplementary material

## Data Availability

All data, including the three questionnaires, the FEC data and the species summary data, have been anonymised and placed in a University of Glasgow repository under a CC‐BY licence and can be accessed at http://doi.org/10.5525/gla.researchdata.1240
