# Supplementary material for: Moxidectin use in Scottish sheep flocks suggests a need for clearer product labelling and communication of updated SCOPS guidelines
Source: Vet Rec. 2022 Aug 27;192(2):e2083. doi: 10.1002/vetr.2083 (PMC10087414; doi:10.1002/vetr.2083)
Supplement: Supplementary file 1 — Supplementary material [file VETR-192-no-s002.pdf]

This questionnaire is part of a research project by Glasgow University and Flock Health Ltd called 'WORMSS', in association with Queen's University Belfast and the Moredun Research Institute. It's about parasite control on sheep farms. We would like to know what you are doing to control worms, and what you think of wormer resistance and how to deal with it.

If you have already completed the online WORMSS survey – THANK YOU – we do not need you to fill out this questionnaire.

There are 9 questions and we estimate that the survey will take around **5 minutes** to complete.

Anonymised answers will be shared between WORMSS and Queen's University Belfast and stored securely in accordance with current Data Protection policies. After the project finishes answers will be stored in a **completely anonymised form** for 10 years, in University of Glasgow archiving facilities in accordance with relevant Data Protection policies and regulations. Questionnaire data, excluding any personal data, may be used by others in the future.

You are under no obligation to take the survey. Results will be used to assess the current situation with respect to wormer resistance, and to improve guidance to help address this issue. They will not be shared with commercial companies except in published form open to everyone.

If you have any questions or concerns about this survey feel free to contact me at Jennifer.mcintyre@glasgow.ac.uk. Thank you in advance for completing this questionnaire!

---

1. Pease confirm, by ticking the box below, that you understand that once you have completed the survey, the question answers will be used for research purposes and the details you have shared cannot be retracted.

☐ I consent to the details that I have provided being used for research purposes under the conditions set out above, and that I cannot retract the information at a later stage.

2. Please enter your county ……………………………………………………………………………………………………….

3. How many breeding ewes do you keep approximately?

☐ Fewer than 50
☐ 50-199
☐ 200-399
☐ 400-999
☐ 1000 or more

4. When did you treat your sheep with moxidectin in 2020? Please fill out the table for all classes/ages of sheep in your flock, as this will provide us with really useful data for our project.

We would like to know:

a. The age/class of sheep (e.g. lambs, ewes, other adults etc.)
b. The reason for treatment (e.g. Lambing, worming, quarantine, tupping, housing, scab, fluke etc.)
c. The approximate percentage of animals in the group treated (e.g. 100%, 90% - 99% etc)
d. The moxidectin formulation (Oral drench, 1% injection, 2% LA injection, Triclamox)
e. The month

Sheep age	Product used	Reason	Percentage treated	Month treated
e.g. Ewes	*Oral drench*	*Lambing*	*90%*	*March*
e.g. All sheep	*Triclamox*	*Fluke*	*100%*	*November*

Sheep age	Product used	Reason	Percentage treated	Month treated

5. Do you think that lambs do better if you treat their ewes with a wormer at lambing time?
- ☐ Yes, if moxidectin
- ☐ Yes, any wormer works equally well
- ☐ Depends on other factors
- ☐ Not sure
- ☐ No

6. Do you intend to change your overall use of moxidectin in the future?
- ☐ Increase use
- ☐ Decrease use
- ☐ No change

7. **If** you use a class 4 (orange) or 5 (purple) wormer, under what circumstances (tick all that apply)?
- ☐ As my main wormer in ewes or lambs, as specified above.
- ☐ Late in the grazing season, as a 'break drench' to eliminate build-up of worms resistant to my main wormer.
- ☐ A few weeks after using moxidectin (group 3), as a 'tailcutter' dose, targeting worms that might develop moxidectin resistance
- ☐ Inserted into my wormer rotation in other ways
- ☐ As a salvage dose when other wormer does not have the desired effect
- ☐ It is my only choice as classes 1-3 no longer work

8. Do you think all of the anthelmintics (wormers) are working effectively in sheep on your farm?
- ☐ Yes
- ☐ No

9. If any products seem to be not working effectively, which (tick all that apply)?
For more information on wormer groups see the SCOPS anthelmintic guide
- ☐ Group 1 (white)
- ☐ Group 2 (yellow)
- ☐ Group 3 (clear)
- ☐ Group 3 (clear: moxidectin)
- ☐ Group 4 (orange)
- ☐ Group 5 (purple)
- ☐ Flukicide: triclabendazole
- ☐ Flukicide: other

Thank you very much for completing this questionnaire! We will use the results to better understand worm control practices on sheep farms, and where they might be improved. Your time is appreciated.

If you would like more information about the WORMSS project, looking at wormer resistance and moxidectin use on Scottish sheep farms, please visit bugconsortium.wordpress.com/category/blog/. We are looking for farmers to submit sheep poo, and receive faecal egg counts in return.

Kindly sponsored by the KTN, via the BSAS Steve Bishop Early Career Award
