# Supplementary material for: Moxidectin use in Scottish sheep flocks suggests a need for clearer product labelling and communication of updated SCOPS guidelines
Source: Vet Rec. 2022 Aug 27;192(2):e2083. doi: 10.1002/vetr.2083 (PMC10087414; doi:10.1002/vetr.2083)
Supplement: Supplementary file 2 — Supplementary material [file VETR-192-no-s001.pdf]

Thank you very much for giving up your time to aid in this research project. We will process your samples and return back to you the strongyle faecal egg count results as quickly as possible.

For all multiple-choice questions please tick or circle the correct answer. Thank you!

Faecal sample details:

1. Date of moxidectin treatment (if your **sampled ewes** were treated over several days please specify the range of dates): ………………………………………………………..

2. Date of faecal sample collection: ………………………………………………………

3. Which moxidectin product did you use? ……………………………………………………………….

4. Roughly when did you last calibrate:

	**Your dosing gun**	**Your weigh scales**
Immediately before use on this occasion		
Within the last month		
Within the last year but not within the last month		
Longer than a year ago		
Never		
Don’t use		

5. How did you collect the faecal samples?

   - ☐ From the field
   - ☐ From a yard or housing area
   - ☐ From the rectum

6. To your knowledge, had the ewes that you sampled been treated with moxidectin before during their lifetime?

   - ☐ All of them, within the last year
   - ☐ All of them, longer than a year ago
   - ☐ Only some of them had been treated before now
   - ☐ No
   - ☐ Don’t know

Please carefully check the sample collection and submission guidelines.

Did you process the samples EXACTLY as required? If not (and we know real life is rarely simple) what did you do differently? We need to know so that we can process your samples correctly in the lab. If you want to check anything please contact Jennifer McIntyre.

☐ Yes, I processed samples as described on the sample collection form.
☐ No – please state what you did differently ……………………………………………………………….
………

Kindly sponsored by the KTN, via the BSAS Steve Bishop Early Career Award

To enable us to better understand your results within our research project, we would appreciate it if you could provide as many details as you can for the following questions. All will help us to meet our project aims and will provide information relevant to interpretation of the faecal samples.

General worm management questions (please tick or circle the answers that apply to your farm)

1. How many breeding ewes do you keep approximately?

    - ☐ Fewer than 50
    - ☐ 50-199
    - ☐ 200-399
    - ☐ 400-999
    - ☐ 1000 or more

2. Do you buy in replacement ewes? ... Yes / No

3. If you buy in replacement sheep (including tups), what do you use as a quarantine treatment for worms? (For more information on wormer groups see the SCOPS anthelmintic guide)

    - ☐ I don't buy in any sheep
    - ☐ Group 1 (white)
    - ☐ Group 2 (yellow)
    - ☐ Group 3 (clear)
    - ☐ Group 3 (clear - moxidectin)
    - ☐ Group 4 (orange)
    - ☐ Group 5 (purple)
    - ☐ I don't treat for worms

4. Do your sheep graze pasture that is grazed by other flocks?

    - ☐ Yes
    - ☐ No
    - ☐ Only during some years

5. How many times per year do you **typically** treat **ewes** for roundworms, on average?

    - ☐ 0
    - ☐ 1
    - ☐ 2
    - ☐ 3
    - ☐ 4 or more

6. How many times per year do you **typically** treat **other adult sheep** for roundworms, on average?

    - ☐ 0
    - ☐ 1
    - ☐ 2
    - ☐ 3
    - ☐ 4 or more

7. How many times per year do you **typically** treat your **lambs** for roundworms?

   - ☐ 0
   - ☐ 1
   - ☐ 2
   - ☐ 3
   - ☐ 4
   - ☐ 5
   - ☐ 6
   - ☐ 7 or more

8. Approximately how many times have you used a 3-ML clear drench or injectable product (**ivermectin products, Eprinex or Dectomax BUT NOT moxidectin)** in your flock over the last 5 years? …………………………………………………………

Now we would like to learn more about how you use moxidectin (Cydectin/Moxodex/Triclamox) in general on your farm.

1. For how long have you been using moxidectin on your farm (for any reason)?
   - ☐ This is the first time I've ever used it
   - ☐ First used it last year
   - ☐ Started using it longer than a year ago but within the last 5 years (2015 onwards)
   - ☐ Started using it more than 5 years ago (before 2015)

2. Do you ever use a 'tailcutting' dose? – (this means that you use a different worming treatment towards the end of the moxidectin persistency period) … Yes / No

Your moxidectin use from January 2020 to December 2020:

1. If you used moxidectin last year (2020), on how many separate occasions did you use it overall on your farm (all sheep, goats and cattle)?
   ……………………………………………………………………………

2. When did you treat your **sheep** with moxidectin **in 2020**? Please fill out the table for all classes/ages of sheep in your flock, as this will provide us with really useful data for our project. It will also help us better interpret your faecal sample data.

   We would like to know:

   a. The age/class of sheep (e.g. lambs, ewes, non-pregnant adults etc)

   b. The moxidectin formulation (Oral drench, 1% injection, 2% LA injection, Triclamox)

   c. The reason for treatment (e.g. Lambing, worming, quarantine, tupping, housing, scab, fluke etc.)

   d. The approximate percentage of animals in the group treated (e.g. 100%, 90% - 99% etc)

   e. The month

Sheep age	Product used	Reason	Percentage treated	Month treated
e.g. Ewes	*Oral drench*	*Lambing*	*90%*	*March*
e.g. All sheep	*Triclamox*	*Fluke*	*100%*	*November*

Your moxidectin use at LAMBING THIS YEAR (2021). If you have separate lambing groups, which you treat differently, **we would like to know about the group containing the ewes you sampled**. If you have not yet finished lambing, please answer with your intention for the whole period of lambing:

1. At **lambing THIS year**, what proportion of ewes did you treat? …………………………………..
2. Were your ewes housed when treated? … Yes / No (Go to Q 4)
3. If housed when treated, had you also treated the ewes at the start of the housing period?
   - ☐ Yes – I treated them with ……………………………………………..
   - ☐ No, I didn't treat them
4. When you move ewes to new fields after lambing, do you move them to:
   - ☐ 'Clean' fields, expected to have low numbers of worms
   - ☐ 'Dirty' fields, expected to have high numbers of worms
   - ☐ Fields which are classed as neither 'clean' nor 'dirty'
   - ☐ Fields with an unknown level of contamination
5. When you put ewes to new fields after lambing do you mix moxidectin treated and untreated ewes in the same field?
   - ☐ Yes, in all fields
   - ☐ In some fields only
   - ☐ No, I keep treated and untreated ewes separate
   - ☐ I treat all my ewes
6. Do you have fields containing both moxidectin treated ewes that lambed at the start of lambing AND treated ewes that lambed towards the end of the lambing period? … Yes / No

Other questions concerning moxidectin use:

1. Do you intend to use moxidectin in your flock next year (2022)?
   - ☐ Same as this year
   - ☐ Different to this year - please specify
     ………..
   - ☐ Don't plan to use
2. Do you intend to change your overall use of moxidectin in the future?
   - ☐ Increase use
   - ☐ Decrease use
   - ☐ No change
3. Do you think that lambs do better if you treat their ewes with a wormer at lambing time?
   - ☐ Yes, if moxidectin
   - ☐ Yes, any wormer works equally well
   - ☐ Depends on other factors
   - ☐ Not sure
   - ☐ No

Wormer resistance (Anthelmintic Resistance)

1. Do you think all of the anthelmintics (wormers) are working effectively in sheep on your farm? ... Yes / No

2. If any products seem to be not working effectively, which (tick all that apply)? (For more information on wormer groups see the SCOPS anthelmintic guide)
    - ☐ Group 1 (white)
    - ☐ Group 2 (yellow)
    - ☐ Group 3 (clear)
    - ☐ Group 3 (clear - moxidectin)
    - ☐ Group 4 (orange)
    - ☐ Group 5 (purple)

3. If you think that any wormers are **not** working properly in your sheep, why do you think this (tick all that apply)?
    - ☐ Full faecal egg count reduction test: run through vet and using individual samples
    - ☐ Drench check: faecal worm egg count before and after treatment using pooled group samples
    - ☐ Drench check but using post-treatment samples only
    - ☐ Sheep not doing well or scouring in spite of treatment
    - ☐ Having to treat more often than expected

4. Please indicate how concerned you are about **clear drench or injectable** wormer resistance in your flock (NOT including moxidectin)?

    Not worried at all -- Very concerned

5. Please indicate how concerned you are about **moxidectin** resistance in your flock?

    Not worried at all -- Very concerned

6. Do you actively try to maintain a ‘refugia’ population of worms on your farm that are sensitive to wormers? … Yes / No

7. In the FUTURE, what would you like to be the MAIN way you manage worms in your flock?

Wormers / anthelmintics	Pasture management	Other livestock / livestock rotation	Breeding	Vaccine	Other

Thank you for taking the time to answer this questionnaire.
