# Supplementary material for: Moxidectin use in Scottish sheep flocks suggests a need for clearer product labelling and communication of updated SCOPS guidelines
Source: Vet Rec. 2022 Aug 27;192(2):e2083. doi: 10.1002/vetr.2083 (PMC10087414; doi:10.1002/vetr.2083)
Supplement: Supplementary file 3 — Supplementary material [file VETR-192-no-s003.docx]

**WORMSS - Faecal sampling protocol**

Faecal sampling pack – please check you have the following:

- Gloves
- Thin plastic bags – at least 15 (one per sheep)
- Ziplock bags
- Padded envelope
- Privacy notice
- Consent form
- Questionnaire

How faeces are collected and stored can make a difference to the faecal egg count. Please follow the instructions below, and contact Jennifer if you have any questions or concerns.

We need you to send back your signed consent form and completed questionnaire with your samples. It is best to complete as much of the information as possible in advance, so that there is no delay in sending the samples to us.

**Most importantly of all – make sure that you are sampling sheep that have been treated with a moxidectin product in the last 14-21 days.** Ideally from sheep treated 17-21 days ago. If you sample sheep that haven’t been treated, or were treated longer than 21 days ago, it can appear as though the wormer hasn’t worked and there is resistance.

1. Put on your gloves!
2. Collect **FRESH** (i.e. still warm) faeces from **15 sheep** into the thin plastic bags – use ONE plastic bag PER sheep.
3. Once you have collected your samples, get the faeces into the bottom of each bag. Leave the top of the bag open (i.e. do not tie closed).
4. Lay the bags flat – one on top of the other, so that the faeces are all at the same side of the pile.
5. Squeeze/press all air out of the bags – it is important that the faeces have as little air as possible, otherwise eggs will start to develop and larvae could hatch, falsely reducing the faecal egg count.
6. Roll all the bags up TIGHTLY, as though they were one large bag.
7. Put the tightly rolled bags into the ziplock bag.
8. Squeeze/press the air out of the ziplock bag and seal it. Put the ziplock bag into the padded envelope.
9. Add your completed questionnaire, consent form and privacy notice, in a second ziplock bag, to the padded envelope.
10. Keep the padded envelope at room temperature (i.e. don’t put it somewhere very hot or cold – like the fridge, or leave it outside for long)
11. Post the samples the same day as collection.

Tips:

- Collect samples in a way you are familiar with.
- If you haven’t collected faeces before, an easy way is to gather your sheep into a corner of the field/bring them indoors – then let them go. As they disperse, gather the fresh (steaming) faeces from the floor. ***** Don’t do this if you have a mixed group of treated and untreated ewes *** Make sure that you only gather treated ewes into the area that you then collect from *****
- If you are collecting from the ground, and the faeces are not pelleted (soft) – try to scoop the faeces up so that you leave behind a thin layer of faecal pat. This reduces the risk of collecting larvae from the grass.
- If you have both ewes and young lambs together – the ewe faeces will be large, while the lambs will most likely be small and pelleted.
- The easiest way to pick up a sample: turn the bag inside out and use the bag like a second glove – pick up the faeces, then ‘peel’ the bag off your arm and over the sample. This will mean that the faeces are at the bottom of the bag.
- If you think that you have too much poo to put all samples easily into a single ziplock bag, then split them into separate bag piles before flattening and rolling up.
- Ideally, we would like a good handful of poo from each sheep – this will allow us to perform a faecal egg count, and culture excess faeces to obtain larvae to identify worm species. We would rather have too much poo than too little!
